# Oculometric Function More Strongly Predicts Working Memory than Stress in Military Officers

**DOI:** 10.3390/jemr19030046

**Published:** 2026-05-02

**Authors:** Mollie McGuire, Neda Bahrani, Quinn Kennedy, Dorion Liston

**Affiliations:** 1 Department of Information Sciences, Naval Postgraduate School, Monterey, CA 93943, USA; mrmcguir@nps.edu; 2 neuroFit, Mountain View, CA 94040, USA; nedabahrani16@berkeley.edu (N.B.); qkennedy@neurofit.tech (Q.K.)

**Keywords:** oculometrics, stress, cognition, military

## Abstract

Working memory, the capacity to store information for near-immediate use, and visual attention, the ability to focus on task-relevant information, are integral skills for military personnel. In civilian populations, stress is associated with worse skills. However, little is known about the relationship between stress, working memory, and visual attention in military officers, who are trained to handle acute stress and operate in high-stress environments. Thirty-three military officers completed a working memory test, a Perceived Stress Questionnaire (PSQ), and an oculometric assessment of visual tracking. The oculometric test was a modified step-ramp test that produces 10 z-scored metrics. Working memory and executive function were assessed via the n-back task. Oculometric performance and self-reported stress levels were independently associated with n-back accuracy, explaining 67% of the variance (adjusted R^2^, *n* = 30). The association between oculometric performance and n-back accuracy was driven by directional anisotropy, directional noise and proportion of smooth pursuit. The association between oculometric performance and stress was complicated by sex differences. Results have important implications for the assessment of cognitive readiness in military populations. The strong relationship between oculometric performance and working memory suggests that eye-tracking-based metrics may serve as candidate indicators of cognitive function under operational demands.

## 1. Introduction

Working memory, the capacity to temporarily store and manipulate information for ongoing cognitive tasks, is a core component of executive function that supports a wide range of behaviors from everyday activities to high-stakes decision-making. In daily life, working memory is essential for tasks such as reasoning, problem-solving, language comprehension, and goal-directed behavior [1,2,3,4]. In more demanding environments, including military and operational settings, working memory underlies complex processes such as multitasking, situational awareness, and rapid decision-making under pressure [5,6]. Impairments or variability in working memory performance have been linked to reduced cognitive efficiency and decreased performance across both laboratory and real-world contexts, highlighting its critical role in adaptive functioning.

**Stress and Cognitive Function**

Stress exerts dynamic and multi-level effects on neural systems supporting executive function, attention, and working memory [7,8,9]. However, the magnitude and direction of these relationships depend critically on how stress is defined and measured. Much of the existing literature relies on experimentally induced acute stress or physiological biomarkers (e.g., cortisol), which capture short-term stress. In contrast, fewer studies have examined how perceived chronic stress, reflecting individuals’ appraisal of ongoing demands over time, relates to baseline cognitive performance. These distinctions are important, as acute and chronic stress may influence cognition through different mechanisms and may show different patterns of association with behavior [10,11,12].

A large body of work provides mechanistic evidence linking stress to cognitive function. Chronic stress has been associated with structural and functional changes in prefrontal and hippocampal systems supporting executive control and working memory [9,11,12,13,14]. At the cellular level, chronic glucocorticoid exposure is associated with changes in synaptic architecture within the prefrontal cortex and hippocampus, alongside impairments in executive control and working memory maintenance [11,12,13]. Converging neuroimaging evidence similarly indicates that stress is associated with disrupted prefrontal processing and altered functional connectivity within executive and attentional networks [12,15,16,17,18]. These findings suggest that chronic stress may alter baseline executive capacity rather than merely perturb performance during acute episodes [13,15].

Together, these findings suggest that stress is broadly linked to variability in executive functioning. However, most of this work focuses on experimentally induced stress or biological measures, leaving open the question of how perceived chronic stress relates to cognitive performance under baseline conditions, particularly in highly trained populations, such as military personnel.

Given these links between stress, attention, and cognitive control, there is increasing interest in identifying behavioral and physiological markers that can capture these processes in real time. At the systems level, stress-related LC–NE activation modulates cortical gain and is indexed peripherally through pupil dilation, a sensitive marker of autonomic arousal [19]. Contemporary neurobiological models link pupillary responses to cortical plasticity and neuromodulatory signaling [20]. In applied contexts, eye-tracking and workload research demonstrate that increased cognitive strain and sustained workload correlate with altered gaze patterns, fixation dynamics, and reduced scanning efficiency [21,22]. Systematic reviews of neurophysiological workload assessment further confirm that cognitive overload is accompanied by measurable ocular and physiological signatures [23]. These findings support the use of oculometric measures as non-invasive indicators of attentional and cognitive processes, which can be examined alongside behavioral performance.

Longitudinal and operational findings extend these laboratory observations. Sustained psychosocial stress and occupational burnout predict alterations in executive regulation and large-scale functional connectivity [17]. In high-demand military and applied settings, stress exposure is associated with working memory variability and cognitive strain under operational load [12,18]. However, prior work has largely focused on performance under stress exposure, rather than baseline associations between perceived stress and cognitive function in trained populations.

Stress-related cognitive effects follow an inverted-U relationship with arousal intensity. Moderate arousal may transiently optimize PFC function, whereas excessive or sustained stress pushes neuromodulatory systems into maladaptive regimes characterized by reduced executive precision and diminished working memory stability [7,8,10,24]. Chronic exposure may therefore narrow the optimal performance window, increasing vulnerability to overload under sustained high-demand conditions.

Taken together, prior work suggests that stress, particularly when prolonged, is associated with variability in executive function and working memory [7,8,9,10,11,12,18]. Because working memory underlies decision-making, multitasking, and prospective memory, variability in this domain may have important consequences in operational settings requiring sustained cognitive performance.

**Oculometrics, Cognitive Function and Stress**

Oculometrics, defined as quantifiable eye movement metrics, map onto distinct cognitive operations [25,26,27,28] and therefore can provide a non-invasive index of cognitive and attentional processes. Pupil diameter indexes mental effort and cortical gain [19,29], fixation duration reflects depth of processing and attentional dwell time [30], and saccadic frequency and amplitude reflect exploratory sampling and environmental scanning efficiency [31]. Smooth pursuit gain indexes sensorimotor precision and sustained attentional engagement [32], while gaze dispersion (scanpath breadth) reflects how broadly an individual samples available visual information [33]. Contemporary systematic reviews confirm that these metrics reliably track cognitive workload across simulated and operational contexts [21,22,23]. Increased workload has been associated with larger pupil diameter, longer fixation durations, reduced saccadic transitions, and narrower gaze dispersion [21,22,23,26]. In surgical simulations, elevated workload and stress have been correlated with prolonged fixations, increased blink variability, and degraded scan efficiency patterns associated with performance degradation [22,26,34].

Because eye movements are tightly coupled to attentional allocation and frontoparietal cortical control systems, alterations in ocular behavior have been used as indicators of shifts in attentional prioritization, arousal regulation, and executive modulation. Prior work suggests that stress has been associated with narrowed attentional focus. Under evaluative or threat-based stressors, individuals tend to allocate gaze toward centrally relevant or salient stimuli while reducing peripheral monitoring. Eye-tracking studies have demonstrated associations with attenuated visual exploration, reduced saccadic frequency, and decreased scanpath breadth under stress exposure [25]. Although many of these findings arise from acute paradigms, chronic stress may similarly be associated with a more persistent form of attentional compression through sustained salience-network bias and reduced executive coordination [7,8].

Reduced scanpath breadth and diminished saccadic transitions under stress have been observed across laboratory paradigms and applied simulations. These ocular changes are particularly consequential for tasks requiring distributed attention and dynamic updating. In aviation simulators, elevated workload has been associated with fewer instrument cross-checks, shorter saccades, and clustered fixations—signatures of attentional tunneling [27,35]. In surgical contexts, stress and fatigue have been linked to saccadic intrusions, degraded smooth pursuit stability, and prolonged fixation durations, indicating impaired visual control under cognitive strain [22,26].

Across domains, the interplay between stress physiology and ocular behavior has been associated with differences in situational awareness. When stress is associated with reduced visual exploration and fewer saccadic transitions, the effective attentional field may contract. Although moderate arousal has been associated with more focused processing, excessive or sustained stress has been linked to reduced executive modulation and more constrained environmental scanning [7,8,12,24]. These patterns have been proposed as one possible mechanism underlying observed associations between stress and working memory performance, particularly in tasks requiring continuous updating and attentional control. In the present study, we examine these relationships at the behavioral level by assessing associations between oculometric performance (nFit), working memory accuracy (n-back), and perceived chronic stress.

**Interplay of stress, ocular function and working memory in military personnel**

The vast majority of the findings described above relate to civilian populations. However, it remains unclear whether the interplay between chronic stress, ocular function, and working memory generalizes to military personnel. Military populations differ from civilians in multiple ways that could influence the relationships among these factors. In particular, military personnel undergo structured stress inoculation training (SIT) and related programs designed to build psychological resilience through controlled exposure to progressively demanding stressors. These programs, often grounded in cognitive–behavioral principles, train individuals to regulate physiological and cognitive responses under conditions such as time pressure, uncertainty, and sensory overload, with the goal of maintaining performance under stress. In high-demand military contexts, stress exposure has been associated with variability in working memory and cognitive performance [18]. More broadly, chronic and repeated stress exposure may shape baseline cognitive and physiological function through adaptive and maladaptive mechanisms within stress-regulation systems [11,12]. These factors may shape baseline cognitive and oculometric performance, as well as how stress is experienced and reported.

To date, the literature on military personnel has largely focused on acute stress paradigms [18]. However, chronic stress is particularly relevant to sustained operational environments [12,17]. Importantly, the present study assesses perceived chronic stress via self-report, rather than acute stress responses, and therefore focuses on baseline individual differences rather than stress reactivity or experimentally induced stress effects.

In sum, while substantial literature has examined stress effects on executive performance and separate bodies of research have characterized stress-related ocular changes, comparatively little work has examined these domains together within the same individuals. Few studies have directly examined how composite oculometric metrics relate simultaneously to working memory performance and perceived stress in highly trained military populations outside clinical frameworks. Most prior work isolates behavioral cognitive outcomes from visual attention metrics, leaving unresolved how real-time ocular signatures correspond to executive functioning under operational conditions.

Addressing this gap is important, as military and other high-performance environments require the coordinated engagement of both executive control and sustained visual monitoring. Oculometric measures may therefore provide a useful, non-invasive index of cognitive and attentional processes that can be examined alongside behavioral performance. In the present study, we investigate associations between oculometric performance (nFit), working memory accuracy (n-back), and perceived chronic stress (PSQ) to better characterize these relationships in a military sample.

**Purpose of the Present Study**

The present study adopts an exploratory, individual-differences approach to examine relationships between oculometric performance, working memory performance, and self-reported chronic stress in a military sample. Specifically, we investigated whether eye-movement-based measures of visual tracking precision, as quantified by the nFit assessment, are associated with working memory accuracy on an n-back task, and whether perceived chronic stress relates to either working memory performance or oculometric performance. In this highly trained military sample, we predicted that nFit would more strongly predict working memory performance than perceived chronic stress and the association between nFit and perceived chronic stress would be modest. By focusing on objective measures of attentional stability and visual control, this work aims to clarify how stress-related cognitive variability, particularly in working memory, manifests in military populations, and to evaluate the potential utility of oculometric metrics as indicators of cognitive readiness under operational demands. The Naval Postgraduate School IRB approved this study (NPS.2024.0015-IR-EP3-4-6-7_A).

## 2. Materials and Methods

As part of a larger stress study, 33 active-duty personnel from four U.S. services (nine Army, four Air Force, five Marines, eleven Navy), and three non-U.S. services (one Australian Air Force, one German Navy, and one Mexican Army) participated. Approximately one half of participants were female. Mean age was 33 years (SD = 4.42). Table 1 provides descriptive statistics on demographic variables.

**Instruments**

Exit Questionnaire

The Exit Questionnaire collected basic participant demographics including age, sex, active-duty status, and occupation, as well as level of engagement during the experiment, and whether the participant ate, drank, smoked, or exercised before the experiment. The questionnaire was given to the participant at the end of the experiment to complete before being debriefed. The purpose of the questionnaire was descriptive.

Chronic stress survey

Chronic stress exposure was assessed using a brief, validated self-report instrument derived from the Perceived Stress Questionnaire (PSQ) [36], a brief, validated, self-report instrument with appropriate internal reliability [37]. Participants rate the frequency of 30 stress-related thoughts and feelings experienced over the past month on a 4-point Likert scale ranging from “almost never” to “usually.” Eight items are listed positively, and their scores are reverse-coded. A PSQ Index (PSQI) is computed by subtracting 30 from the raw score and dividing the result by 90, which yields a score between 0.0 and 1.0. The PSQI is the outcome measure used in analyses.

Oculometric test

The oculometric assessment was delivered on the nFit headset app (neuroFit, Mountain View, CA), a standardized, automated five-minute measure of oculomotor control and attention adapted from the step-ramp tracking task and described in detail by Liston and Stone [28]. The test begins with a nine-point calibration. We calibrated the eye tracker using a standard nine-point calibration and fit the eye position traces for each eye using five parameters (two offsets, two gains, one rotation) which yielded an overall error and a noise level for each calibration. The headset we used offered a GazeRecognition API that identified fixations, blinks, saccades, and other movements. We used the manufacturer-provided signals to isolate and remove blinks from the eye-position traces. Participants were instructed to fixate on the dot until it disappeared. Participants were instructed to visually track a moving dot that followed unpredictable trajectories over 45 trials. The task produced ten *z*-scored oculometric metrics capturing smooth pursuit accuracy, saccadic behavior, and tracking noise, including latency, acceleration, gain, catch-up saccade amplitude, proportion of smooth tracking, direction noise, speed tuning responsiveness, and speed noise. A summary score called nFit was computed by transforming each ocular measurement into a z-score referenced to a previously collected normative control sample [28], averaging the vector while accounting for the covariance. Thus, the formula for nFit yields a normal distribution centered at a mean of 0 and standard deviation of 1 for the normative control population. This transform allows for quick tests of significance and easy conversions into percentiles using the inverse of the normal cumulative distribution function. For example, an nFit score above 1.645 or below −1.645 is significant at the 0.05 level using a one-tailed test, and practicing physicians use nFit of −2.0 as a criterion for TBI detection. nFit scores lower than 0.0 indicate reduced smooth pursuit gain, increased noise, and more saccadic interruptions, patterns consistent with attentional narrowing or “tunnel vision” that can emerge under conditions of physiological compromise including fatigue and acute stress.

Cognitive Test

n-back test

Working memory performance was assessed using a computerized n-back task [5]. Participants were presented with a continuous sequence of stimuli and were instructed to indicate whether the current stimulus matched the one presented *n* positions earlier in the sequence. The test requires continuous updating and maintenance of information in working memory while inhibiting responses to non-target stimuli [38]. It consists of a practice block of 8 trials, followed by three test blocks, 0-back, 1-back, and 2-back, presented in a random order. Each test block consists of 50 trials for a total of 150 trials. For each trial, the participant’s response is automatically classified as a hit, miss, correct rejection, and false alarm. The main performance measure was accuracy, calculated as the proportion of correct responses (hits and correct rejections) of the 150 trials. The n-back test was administered under standardized conditions.

**Equipment**

Augmented Reality (AR) headset

The oculometric assessment was conducted using a Magic Leap 2 AR headset (Magic Leap, Plantation, FL) equipped with an integrated high-resolution eye-tracking system. The headset allows for precise measurement of gaze position, pupil dynamics, and saccadic movements in real time, enabling accurate quantification of visual tracking performance. After launching the oculometric assessment application, the task proceeded in a fully automated manner. Visual graphics and auditory prompts guided participants through each step of the procedure, ensuring standardized task administration across sessions. The headset’s embedded sensors and algorithm stack continuously streamed binocular 2D pupil position and 3D gaze vectors at 60 Hz. Data were automatically uploaded to a secure, encrypted research server following each session. The use of the Magic Leap headset provided an immersive testing environment that minimized external distractions and allowed for consistent calibration across participants.

**Experimental Design**

As part of a larger longitudinal study examining the relationship between stress, cognition, and visual processing ability in active-duty personnel, we analyzed participants’ baseline performance on each measure.

**Procedures**

This study was conducted in a laboratory at the Naval Postgraduate School. Upon arrival for the stress study, participants were greeted by a trained researcher, provided informed consent, and then were asked to rest for five minutes before starting the experiment. This provided time to establish a calm baseline. They were seated, fitted with the headset system, and completed eye-tracking calibration. Next, they performed the oculometric test followed by instructions and practice trials of the n-back test. They then completed the n-back test. Finally, participants were administered the stress scale and an Exit Questionnaire before they were debriefed and thanked for their participation. The task order was fixed for all participants.

**Statistical Analyses**

Descriptive statistics and multivariate correlations were used for preliminary analyses on demographic variables, the main variables, and to determine if oculometric data from each eye could be merged. Primary analyses of the association between nFit, PSQI, and n-back performance were conducted with stepwise regression. Exploratory analyses were conducted on the association between individual ocular metrics and n-back performance, as well as to further understand any demographic differences on the nFit or the PSQI. The Holm–Bonferroni sequential correction method with a two-tailed familywise error rate of 0.05 was applied to multiple comparisons [39]. We used JMP Pro 15 software.

## 3. Results

### 3.1. Preliminary Results

Descriptive statistics revealed that two subjects had nFit scores below −3.00. Excluding the two nFit outliers only modestly changed the mean nFit score from −0.72 (SD = 1.21) to −0.51 (SD = 0.89). One of these subjects also had an outlier n-back accuracy score of 0.84. Again, removing this data point only modestly changed mean n-back accuracy score from 0.947 (SD = 0.037) to 0.951 (SD = 0.030). We conducted the analyses with and without these outliers and found consistent results. Therefore, we report results that include all subjects.

Table 1 provides descriptive statistics on the main measures. Subjects performed significantly worse than 0 on nFit (*t*(30) = −3.30, *p* = 0.003). In contrast, most subjects performed above 90% on the n-back test with half the sample scoring 96% or better across all three blocks. Finally, subjects reported moderate amounts of chronic stress, with female participants reporting significantly higher ratings than male participants (female mean = 0.42, SD = 0.12, male mean = 0.32, SD = 0.13, *t*(8.09) = 2.55, *p* = 0.017). No other sex differences were observed (*p* > 0.20).

Nearly all individual ocular metrics from each eye were strongly correlated (*r* ranging from 0.49 (speed noise) to 0.89 (SS gain); all *n* = 30); see Table 2. Only directional anisotropy (*r* = 0.38, *p* = 0.036) and saccadic amplitude (*r* = 0.35, *p* = 0.058) had modest correlations. These results remained the same after applying the Holm–Bonferroni sequential correction. These correlations were consistent with and without the two nFit outliers. Therefore, for each ocular metric, scores from each eye were averaged together and the averaged ocular metric was used in analyses.

### 3.2. Main Results

**Figure 1:**
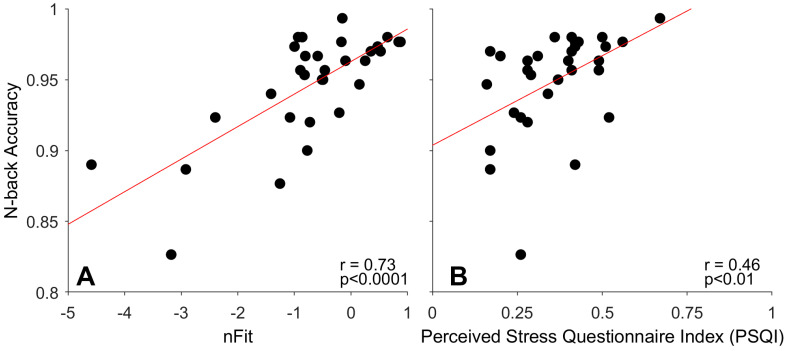
Working memory, nFit, and perceived stress. N-back accuracy correlates with both nFit (**A**) and Perceived Stress Questionnaire index scores (**B**). Each filled circle represents one participant; the solid red line represents the linear regression.

Multivariate correlations indicated that n-back accuracy was positively correlated with nFit (Pearson’s *r* = 0.73, *p* < 0.0001, *n* = 31) and PSQI (Pearson’s *r* = 0.46, *p* < 0.01, *n* = 32) (see Figure 1A,B). Because female participants reported significantly higher PSQI scores than males, we examined the correlation between nFit and PSQI by sex. A multiple regression with PSQI, sex, and PSQI–sex interaction as predictors was not significant (*F*(3,26) = 2.34, RMSE = 1.15, *p* = 0.096). Among females, there was a trend for nFit and PSQI scores to positively correlate (Spearman’s *r* = 0.46, *p* = 0.098; *n* = 14), whereas no pattern emerged for males (Spearman’s *r* = 0.047, *p* = 0.86; *n* = 16).

To further understand the relationship between n-back accuracy, nFit, and PSQI, stepwise regression was conducted using nFit alone, then nFit and PSQI, and finally nFit, PSQI, and nFit x PSQI. In the first regression, nFit explained 52% of the variance (adjusted R^2^) in n-back accuracy (*b*_nFit_ = 0.023, *SE*(*b*_nFit_) = 0.004, *F*(1,29) = 33.93, *p* < 0.0001; *n* = 30). In the second regression, both nFit (*b*_nFit_ = 0.021, *SE*(*b*_nFit_) = 0.003, *t*(27) = 6.39, *p* < 0.0001; *n* = 30) and PSQI (*b*_PSQI_ = 0.098, *SE*(*b*_PSQI_) = 0.03, *t*(27) = 3.25, *p* = 0.003) were significant predictors, accounting for 67% of the variance (adjusted R^2^) in n-back accuracy (*F*(2,27) = 29.98, *p* < 0.0001; *n* = 30). Adding the interaction term in the third regression did not meaningfully change the results. Although the interaction term approached significance (*b*_nFit x PSQI_ = −0.05, *SE*(*b*_nFit x PSQI_) = 0.026, *t*(27) = −2.05, *p* = 0.051), the adjusted R^2^ marginally improved from 67% to 70% (*F*(3,26) = 23.74, *p* < 0.0001; *n* = 30) and the nFit and PSQI estimates and results were nearly identical to those in the second regression (*b*_nFit_ = 0.021, *SE*(*b*_nFit_) = 0.003, *t*(26) = 6.90, *p* < 0.0001; *b*_PSQI_ = 0.102, *SE*(*b*_PSQI_) = 0.029, *t*(26) = 3.57, *p* = 0.001). Finally, a regression with PSQI as the sole predictor accounted for 18% of the variance in n-back accuracy (*b*_PSQI_ = 0.126, *SE*(*b*_PSQI_) = 0.045, *t*(30) = 2.81, *p* = 0.009).

Next, we explored whether individual ocular metrics were driving the association between nFit and n-back accuracy. After applying the Holm–Bonferroni sequential correction for multiple comparisons [39], six ocular metrics were significantly associated with n-back accuracy: directional noise (*r* = −0.67, *p* < 0.0001; *n* = 30), directional anistropy (*r* = −0.56, *p* = 0.001; *n* = 30), directional asymmetry (*r* = −0.43, *p* = 0.018; *n* = 30), initial latency (*r* = −0.53, *p* = 0.003; *n* = 30), proportion smooth (*r* = 0.44, *p* = 0.016; *n* = 30), and speed slope (*r* = 0.40, *p* = 0.027; *n* = 30). However, when the nFit outliers were removed, directional noise (*r* = −0.54, *p* = 0.003; *n* = 28), directional anistropy (*r* = −0.46, *p* = 0.013; *n* = 28), and proportion smooth (*r* = 0.41, *p* = 0.029; *n* = 28) remained significant after the Holm–Bonferroni sequential correction. Thus, there was a consistent pattern in which better directional performance (i.e., smaller errors) and faster eye movement speed predicted n-back accuracy.

## 4. Discussion

The present study examined associations among oculometric performance, working memory, and perceived chronic stress in a sample of active-duty military officers. Several key findings emerged. First, participants demonstrated uniformly high performance on the n-back working memory task, contrasting with comparatively poor performance on the oculometric nFit assessment. Second, n-back accuracy was strongly associated with both nFit performance and perceived chronic stress, with oculometric measures accounting for a substantial proportion of variance in working memory performance. Finally, exploratory analyses revealed sex-related differences in perceived stress and a non-significant trend toward a sex-by-stress interaction in the relationship between perceived stress and oculometric performance. These findings should be interpreted within an exploratory individual-differences framework, reflecting baseline variability.

Officers in the present sample performed exceptionally well on the n-back task, with mean accuracy exceeding 94%. This finding is consistent with the centrality of working memory processes, such as information updating, monitoring, and cognitive control, in military training and operational environments. Working memory capacity is critical for tasks including command-and-control, threat assessment, multitasking, and rapid decision-making under dynamic conditions. Repeated exposure to cognitively demanding situations may therefore confer both selection and training advantages for working memory performance in officer populations. Rather than indicating causal effects of training or selection, these results likely reflect characteristics of the sampled population and task context.

The central finding of this study was the robust association between nFit performance and n-back accuracy. Across analyses, oculometric performance alone explained over half of the variance in working memory accuracy, and together with perceived stress accounted for up to two-thirds of the variability in n-back performance. These results suggest that eye-movement-based measures of visual tracking precision and attentional stability are associated with higher-order cognitive function in military personnel. However, given the cross-sectional and correlational design, these findings do not establish directional or causal relationships between oculometric performance and working memory.

Interestingly, perceived chronic stress was positively associated with n-back performance, a pattern that may appear counterintuitive given extensive literature linking stress to cognitive impairment. However, accumulating evidence suggests that moderate levels of stress have been associated with enhancements in certain aspects of cognitive performance, particularly in well-trained populations, potentially via increased arousal, task engagement, or motivational focus [10,24]. In the present study, this interpretation should be approached with caution, as perceived stress was assessed via self-report and may capture subjective workload, engagement, or responsibility rather than physiological stress per se. In this context, perceived stress may index differences in perceived cognitive demand or engagement rather than a direct facilitative effect on working memory performance.

A notable result was that while oculometric performance and perceived stress each predicted working memory performance, they were uncorrelated to each other. One possible explanation is that the present sample consisted exclusively of officers, for whom stress may be more tightly coupled to responsibility, leadership demands, and cognitive engagement rather than physical threat exposure. Another possibility is that more basic functions, like eye movements, are less impacted by stress than higher level functions, such as working memory. These interpretations are speculative and not directly tested in the present study.

The presence of a sex difference in perceived chronic stress should be interpreted with caution. Although female participants reported higher stress levels than males, no significant differences were observed in oculometric or cognitive performance. Moreover, while there was a trend toward a positive association between perceived stress and nFit performance among female participants, the interaction between sex and stress was not statistically significant. As such, these observations should be considered exploratory and hypothesis-generating rather than conclusive. Prior work has suggested that stress may differentially influence cognitive and affective processes across sexes [40,41], but the present study was not designed to directly test these mechanisms. Future studies with larger samples and targeted designs will be necessary to more rigorously evaluate potential sex differences in the relationship between stress, oculometric performance, and working memory.

Several limitations should be considered when interpreting these findings. First, participants were aware that they were enrolled in a broader stress study involving multiple acute stressors, which may have influenced baseline oculometric performance and contributed to overall lower nFit scores. Anticipatory stress or heightened arousal could plausibly have impacted eye-movement behavior even in the absence of a causal stress manipulation. Second, the cross-sectional design precludes causal inference regarding the relationships among perceived stress, oculometric performance, and working memory. Third, the sample size was modest, limiting statistical power for detecting interaction effects and constraining generalizability. Fourth, the high accuracy observed in the n-back task suggests a potential ceiling effect, which may reduce sensitivity to individual differences in working memory performance. Fifth, perceived stress was assessed using a self-report measure, and no objective physiological or biological markers of stress (e.g., cortisol or heart rate variability) were included.

Despite these limitations, the present findings provide preliminary evidence of associations relevant to the assessment of cognitive performance in military populations. The strong relationship between oculometric performance and working memory suggests that eye-tracking-based metrics may serve as candidate indicators of cognitive function under operational demands. Moreover, the differential associations observed with perceived stress underscore the need for nuanced models that distinguish between different forms of stress (e.g., perceived chronic vs. acute stress), particularly across sexes and ranks. However, these implications should be interpreted cautiously given the exploratory and correlational nature of the present study. Future work should examine these relationships longitudinally, incorporate objective physiological stress markers, and explore how oculometric indicators relate to real-world performance outcomes. Together, these efforts may help refine non-invasive tools for monitoring cognitive readiness and resilience in high-stakes environments.

## Figures and Tables

**Table 1:** Descriptive statistics for demographic and main study variables.

Variable	Statistic	N
Age (years)	Mean = 33.56, SD = 4.42	32
Female	15 (46.9%)	32
U.S. Military Service	30 (91%)	
non-U.S. Military Service	3 (9%)	
nFit	Mean = −0.72, SD = 1.21	31
PSQI	Mean = 0.36, SD = 0.13	32
N-back accuracy	Mean = 0.95, SD = 0.04	33

**Table 2:** Individual oculometric correlations between right and left eyes.

Ocular Metric	Left Eye Mean (SD)	Right Eye Mean (SD)	Left–Right Eye Correlation, *r* (*p*)
Initial latency	174.60 (16.81)	171.40 (9.01)	0.56 (0.0013)
Initial acceleration	170.10 (67.31)	156.50 (47.59)	0.58 (<0.0001)
Smooth pursuit gain	0.62 (0.16)	0.65 (0.16)	0.89 (<0.0001)
Saccade amplitude	1.75 (0.66)	1.80 (0.62)	0.35 (0.0588)
Proportion smooth	0.60 (0.19)	0.63 (0.15)	0.74 (<0.0001)
Directional anisotropy	0.28 (0.68)	0.23 (0.45)	0.38 (0.0363)
Directional asymmetry	0.43 (0.49)	0.34 (0.30)	0.71 (<0.0001)
Directional noise	15.66 (11.70)	11.37 (3.35)	0.59 (<0.0001)
Speed slope	0.21 (0.46)	0.21 (0.44)	0.52 (<0.0001)
Speed noise	5.01 (1.76)	4.47 (2.05)	0.49 (<0.0001)

Note. Each *r* value reflects the Pearson correlation for the left and right eye metrics across all subjects.

## Data Availability

Data available on request due to privacy restrictions related to subject identifiability.

## Abbreviations

The following abbreviations are used in this manuscript:

PFC	Prefrontal cortex
IRB	Institutional Review Board
SD	Standard deviation
PSQ	Perceived Stress Questionnaire
PSQI	Perceived Stress Questionnaire Index
AR	Augmented reality
